# Choosing neoadjuvant therapy for muscle-invasive bladder cancer based on efficacy-safety trade-off: a network meta-analysis

**DOI:** 10.3389/fonc.2026.1819312

**Published:** 2026-06-12

**Authors:** Zilin Zhao, Xinyu Liu, Qikai Zhong, Lin Liu, Runze Pan, Junzhi Chen, Xinlei Wang, Xing Liu, Jiangshang Bao, Yufeng Sun, Conghui Han, Zhenduo Shi, Qing Liang

**Affiliations:** 1 Xuzhou Clinical College of Xuzhou Medical University, Xuzhou, Jiangsu, China; 2 Department of Urology, Xuzhou Central Hospital, Southeast University, Xuzhou, Jiangsu, China; 3 Southeast University, Nanjing, Jiangsu, China

**Keywords:** Bayesian analysis, chemoimmunotherapy, immunotherapy, muscle-invasive bladder cancer, neoadjuvant therapy, network meta-analysis

## Abstract

**Introduction:**

Muscle-invasive bladder cancer (MIBC) carries a poor prognosis, and the optimal neoadjuvant treatment strategy remains debated. This study aimed to systematically compare the efficacy and safety of neoadjuvant chemoimmunotherapy (NICT), chemotherapy alone (NAC), and immunotherapy alone (IT) for MIBC.

**Methods:**

A Bayesian network meta-analysis was conducted. We systematically searched PubMed, Embase, Cochrane Library, and Web of Science for relevant randomized controlled trials and prospective/retrospective cohort studies published up to June 2025. The primary outcomes were pathological complete response (pCR) rate and the incidence of grade ≥3 treatment-related adverse events (TRAEs). Data analysis was performed using R software with appropriate packages for network meta-analysis.

**Results:**

Fourteen studies involving 2234 patients were included. The pooled pCR rate was highest for NICT (0.41, 95% CI: 0.37-0.46), followed by IT (0.26, 95% CI: 0.17-0.39) and NAC (0.15, 95% CI: 0.10-0.23). Network meta-analysis showed NICT was significantly superior to NAC for pCR (OR = 4.2, 95% CI: 2.0-8.2). For grade ≥3 TRAEs, the pooled incidence was highest for NAC (0.19, 95% CI: 0.07-0.39), followed by NICT (0.12, 95% CI: 0.09-0.18) and IT (0.06, 95% CI: 0.04-0.10), with no statistically significant pairwise differences. Ranking probabilities indicated NICT had the highest likelihood of being the most efficacious treatment but also the highest probability of adverse events.

**Conclusion:**

NICT demonstrates the best efficacy in the neoadjuvant treatment of MIBC but is associated with relatively higher toxicity, making it suitable for patients with good cisplatin tolerance. However, the analysis is limited by the lack of long-term survival outcomes such as overall survival and recurrence-free survival. IT offers a safer profile with meaningful efficacy and represents a viable option for patients with poor toxicity tolerance. Treatment selection should be individualized based on efficacy-safety trade-offs and patient-specific factors.

**Systematic Review Registration:**

https://www.crd.york.ac.uk/PROSPERO/view/CRD420251171346, identifier CRD420251171346.

## 1 Introduction

Muscle-invasive bladder cancer (MIBC) has a poor overall prognosis, accounting for 20-30% of bladder cancer cases. Optimizing its neoadjuvant treatment strategy remains a clinical challenge (1). The current standard of care for neoadjuvant setting remains cisplatin-based chemotherapy (NAC) combined with radical cystectomy; however, the 5-year survival rate for MIBC remains below 50%, dropping to <30% in advanced stages (2, 3). Notably, the treatment landscape for MIBC is evolving rapidly. The combination of enfortumab vedotin (EV, an antibody-drug conjugate) plus pembrolizumab has recently demonstrated compelling efficacy in the perioperative setting. The phase III KEYNOTE-B15 trial evaluated neoadjuvant (4 cycles) and adjuvant (5 cycles EV + 13 cycles pembrolizumab) EV plus pembrolizumab versus NAC in cisplatin-eligible patients with MIBC. At a median follow-up of 33.6 months, the EV + pembrolizumab arm significantly improved pathological complete response (pCR) rate (55.8% vs. 32.5%; p<0.0001), event-free survival (HR 0.53), and overall survival (HR 0.65) compared with NAC (4). This regimen is thus emerging as a new standard of care in the perioperative management of MIBC, although not yet incorporated into major guidelines (e.g., NCCN) at the time of this literature search. For advanced or metastatic disease, the standard of care remains EV + pembrolizumab based on the KEYNOTE-302 trial (5). Moreover, the use of NAC is limited by significant toxicity, with up to 50% of patients experiencing grade ≥3 adverse events (AEs). Another 30%-40% of cisplatin-eligible patients discontinue treatment due to renal impairment, myelosuppression, or other complications (6–8). Treatment options remain scarce for patients ineligible for cisplatin.

The advent of immune checkpoint inhibitors (ICIs) has reshaped the treatment landscape for advanced urothelial carcinoma. PD-1/PD-L1 inhibitors (e.g., pembrolizumab, atezolizumab) have shown promising activity in the neoadjuvant setting, with pathological complete response (pCR) rates reaching 15%-30% (9, 10). Nevertheless, the efficacy of monotherapy remains suboptimal, prompting the exploration of combination strategies. Recent evidence suggests chemotherapy combined with immunotherapy (chemoimmunotherapy, NICT) has emerged as a potential breakthrough, with recent trials reporting pCR rates of 40%-50% – significantly higher than with NAC or immunotherapy (IT) alone (11–13). For instance, the BLASST-1 trial demonstrated that nivolumab combined with GC chemotherapy achieved a pCR rate of 43%, significantly superior to the 27% rate with GC chemotherapy alone (14, 15), highlighting the potential of combination strategies.

Existing studies, however, lack a systematic comparison of NICT, NAC, and IT, particularly regarding the balance between efficacy and safety. Previous meta-analyses have focused primarily on pairwise comparisons or single treatment modalities, without simultaneously integrating and ranking all three main neoadjuvant strategies in a unified framework that explicitly quantifies the efficacy-safety trade-off (16–18). While these studies have provided valuable insights, our analysis offers several distinct contributions: first, the inclusion of the recently published phase III NIAGARA trial provides the most up-to-date evidence (19); second, our Bayesian framework simultaneously ranks all three treatment strategies; and third, we explicitly quantify the efficacy-safety trade-off to guide clinical decision-making, which has not been systematically addressed in prior meta-analyses.

This study therefore conducted a Bayesian network meta-analysis (NMA) to integrate direct and indirect evidence, systematically comparing for the first time the efficacy (pCR rate) and safety (grade ≥3 treatment-related adverse events, TRAEs) of NICT, NAC, and IT in the neoadjuvant treatment of MIBC, to guide individualized treatment decisions.

## 2 Methods

### 2.1 Study design and protocol registration

This systematic review and Bayesian network meta-analysis (NMA) was prospectively registered on PROSPERO (registration number CRD420251171346). The conduct and reporting adhered to the Preferred Reporting Items for Systematic Reviews and Meta-Analyses extension for Network Meta-Analyses (PRISMA-NMA) guidelines (20).

### 2.2 Participants

The study population comprised patients diagnosed with muscle-invasive bladder cancer (MIBC) who were scheduled for and subsequently underwent radical cystectomy following neoadjuvant therapy.

### 2.3 Interventions and comparators

Three neoadjuvant treatment strategies were systematically compared:

Experimental Intervention: Neoadjuvant chemoimmunotherapy (NICT), defined as platinum-based chemotherapy combined with a PD-1/PD-L1 inhibitor.

Control Comparators: (1) Neoadjuvant chemotherapy alone (NAC), defined as platinum-based chemotherapy. (2) Neoadjuvant immunotherapy alone (IT), defined as a PD-1/PD-L1 inhibitor monotherapy.

### 2.4 Search strategy

A systematic literature search was conducted across four electronic databases: PubMed, Embase, Cochrane Library, and Web of Science, from their inception until June 2025. The search strategy combined Medical Subject Headings (MeSH) and free-text terms related to “muscle-invasive bladder cancer,” “neoadjuvant,” “immunotherapy,” “chemotherapy,” and relevant study designs. Key search terms included: “muscle-invasive bladder cancer” AND (“neoadjuvant chemotherapy” OR “neoadjuvant”) AND (“immunotherapy” OR “PD-L1” OR “PD-1”) AND (“randomized controlled trial” OR “prospective” OR “retrospective”).

### 2.5 Study selection and data extraction

Two investigators (Z.L.Z. and Q.K.Z.) independently screened titles, abstracts, and full texts against the pre-defined eligibility criteria. Any disagreements were resolved through discussion with a third reviewer (Z.D.S.). Data were extracted using a standardized form. Extracted information encompassed: first author, publication year, study design, sample size, patient baseline characteristics (e.g., age, gender, clinical stage, cisplatin eligibility), details of the intervention regimen (drug names, doses, cycles), and outcome data (number of patients achieving pathological complete response [pCR] and number experiencing grade ≥3 treatment-related adverse events [TRAEs]).

### 2.6 Risk of bias assessment

The risk of bias in included randomized controlled trials (RCTs) was assessed using the Cochrane Risk of Bias tool (RoB 2.0) (21). For non-randomized studies (NRS), the Risk Of Bias In Non-randomized Studies - of Interventions (ROBINS-I) tool was employed (22).

### 2.7 Data analysis

Pairwise meta-analysis was performed using R software (version 4.4.0) with the *meta* and *meta for* packages. Odds ratios (OR) with 95% confidence intervals (CI) were calculated for dichotomous outcomes. Heterogeneity was quantified using the I² statistic, with values of <25%, 25–50%, and >50% indicating low, moderate, and high heterogeneity, respectively (23). A random-effects model was used if significant heterogeneity was present (P < 0.05 or I² > 50%); otherwise, a fixed-effects model was applied. Publication bias was assessed using funnel plots and Egger’s test.

Subsequently, a Bayesian NMA was conducted using the *gemtc*, *rjags*, and *netmeta* packages in R. A network graph was constructed to visualize treatment comparisons. A random-effects consistency model was fitted using Markov Chain Monte Carlo simulation with 4 chains, 5,000 adaptation iterations, and 20,000 sampling iterations. Model convergence was assessed using the Gelman-Rubin statistic (Rê ≈1.0 indicated good convergence). Treatment effects are reported as OR with 95% credible intervals (CrI). The node-splitting method was used to assess local inconsistency (24). Treatment rankings were derived using the surface under the cumulative ranking curve (SUCRA) values, where a higher SUCRA score indicates a greater probability of being the best (or worst) treatment for a given outcome (25). Given the inclusion of non-randomized studies, we planned to perform a sensitivity analysis by excluding these studies to assess their impact on the pooled effect estimates for pCR and TRAEs.

## 3 Results

### 3.1 Study selection and basic characteristics

**Figure 1:**
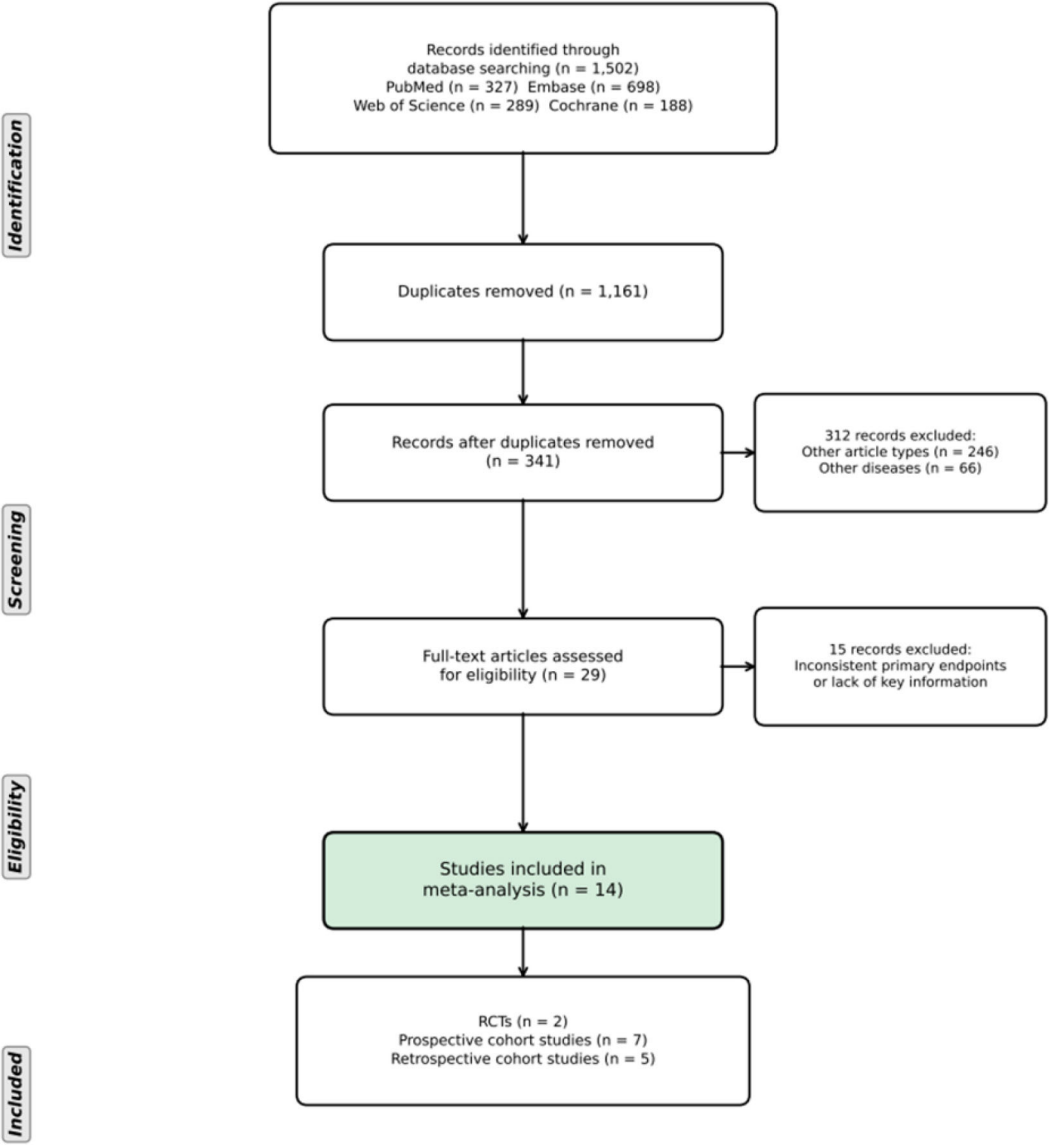
Flowchart of literature search and selection.

Our initial search yielded 1,502 records. After removing 1,161 duplicates, 341 titles and abstracts were screened. We excluded 312 irrelevant records and 15 records lacking key information or consistent primary endpoints. Finally, 14 studies (11–13, 19, 26–35), involving 2234 patients were included. The detailed study selection process is shown in the PRISMA flow diagram (**Figure 1**).

Among the 14 studies, 2 were randomized controlled trials (27, 29), 7 were prospective cohort studies (12, 19, 26, 28, 32–34), and 5 were retrospective cohort studies (11, 13, 30, 31, 35). The median sample size per group was 39 (range: 12 - 194). Most studies used gemcitabine as the chemotherapy backbone (n = 12) and pembrolizumab/nivolumab as the immune checkpoint inhibitor (n = 10). Cisplatin eligibility was reported in 11 studies: 64.3% (472 of 734) of patients were eligible. Basic characteristics of the included studies are summarized in **Table 1**.

**Table 1:** Basic characteristics of included studies.

Author	Year	treatment	N	Age(median/mean years)	Gender(male, %)	Trail ID	cTNM stage	Study design
Jérémy Blanc (24)	2025	NICT	39	63	77	NCT03674424	cT2-cT4a, cN0-cN1, M0	RCT
Jérémy Blanc	2025	NICT	40	68	72			
Jérémy Blanc	2025	NICT	29	72	93			
Jérémy Blanc	2025	IT	29	74	90			
Xinjia Ding (25)	2025	NAC	71	63	79	NA	cT2-cT4, cN0-cN1, M0	non-RCT
Xinjia Ding	2025	NICT	29	65	76			
Hao Zhang (26)	2025	NAC	194	54	84	NA	cT2-cT4, cN0-cN1, M0	non-RCT
Hao Zhang	2025	NICT	126	55	80			
Chuanao Zhang (27)	2025	NICT	21	68	76	NA	cT2-cT4a, cN0-cN1, M0	non-RCT
Thomas Powles (17)	2024	NICT	533	65	82	NA	cT2-cT4a, cN0-cN1, M0	RCT
Thomas Powles	2024	NAC	530	66	82			
Yanhang Yu (28)	2024	NICT	27	68	78	NA	cT2-cT4a, cN0, M0	non-RCT
Yanhang Yu	2024	NAC	26	67	85			
Jiao Hu (29)	2022	NICT	98	66	82	NA	cT2-cT3+, cN0-cN1, M0	non-RCT
Jiao Hu	2022	NAC	107	75	86			
Jiao Hu	2022	IT	48	63	79			
Hongsik Kim (9)	2022	IT	51	66	84	KCT0003804	cT2-cT4a, cN0-cN1, M0	single-arm
Bernadett Szabados (30)	2021	IT	87	72	86	NCT02662309	cT2-cT4a, cN0-cN1, M0	single-arm
Vadim S Koshkin (31)	2021	IT	20	69	75	NCT02451423	cT2-cT4a, cN0-cN1, M0	single-arm
Tracy L Rose (32)	2021	NICT	39	66	82	NCT02690558	cT2-cT4a, cN0, M0	single-arm
Samuel A Funt (10)	2022	NICT	39	65	85	NCT02989584	cT2-cT4a, cN0, M0	single-arm
Shilpa Gupta (11)	2022	NICT	39	NA	NA	NCT03294304	cT2-cT4a, cN0-cN1, M0	non-RCT
Constance Thibault (33)	2020	IT	12	NA	NA	NCT03549715	cT2-cT4a, cN0-cN1, M0	RCT

### 3.2 Risk of bias assessment results

**Figure 2:**
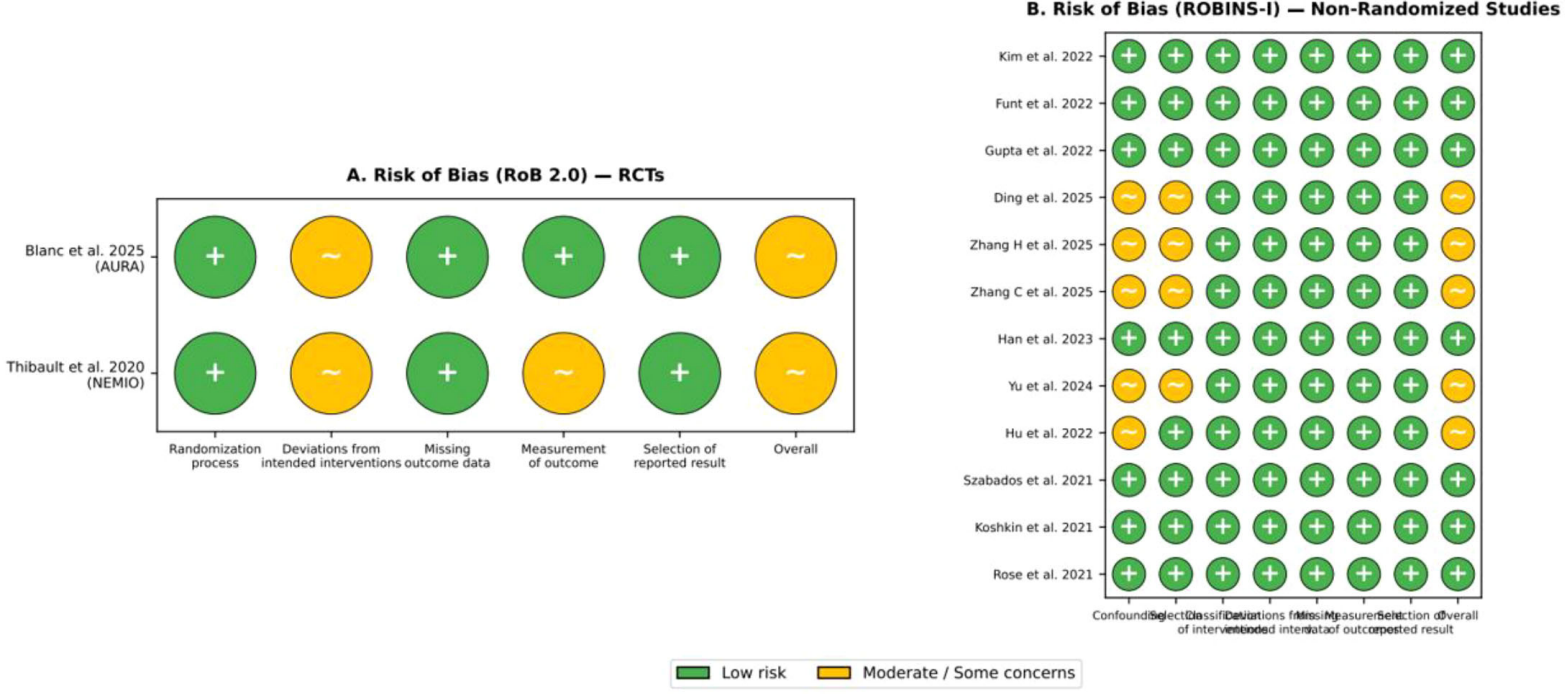
Risk of bias assessment for non-randomized studies using ROBINS-I. **(A)** Summary of bias across studies. **(B)** Individual study ratings for each domain.

The risk of bias for the 2 RCTs was assessed using RoB 2.0, and for the 12 non-randomized studies using ROBINS-I. A summary of the ROBINS-I assessment is presented in **Figure 2**. Overall, most non-randomized studies showed moderate risk of bias, primarily due to confounding and selection of participants.

### 3.3 Meta-analysis of pCR and AEs

**Figure 3:**
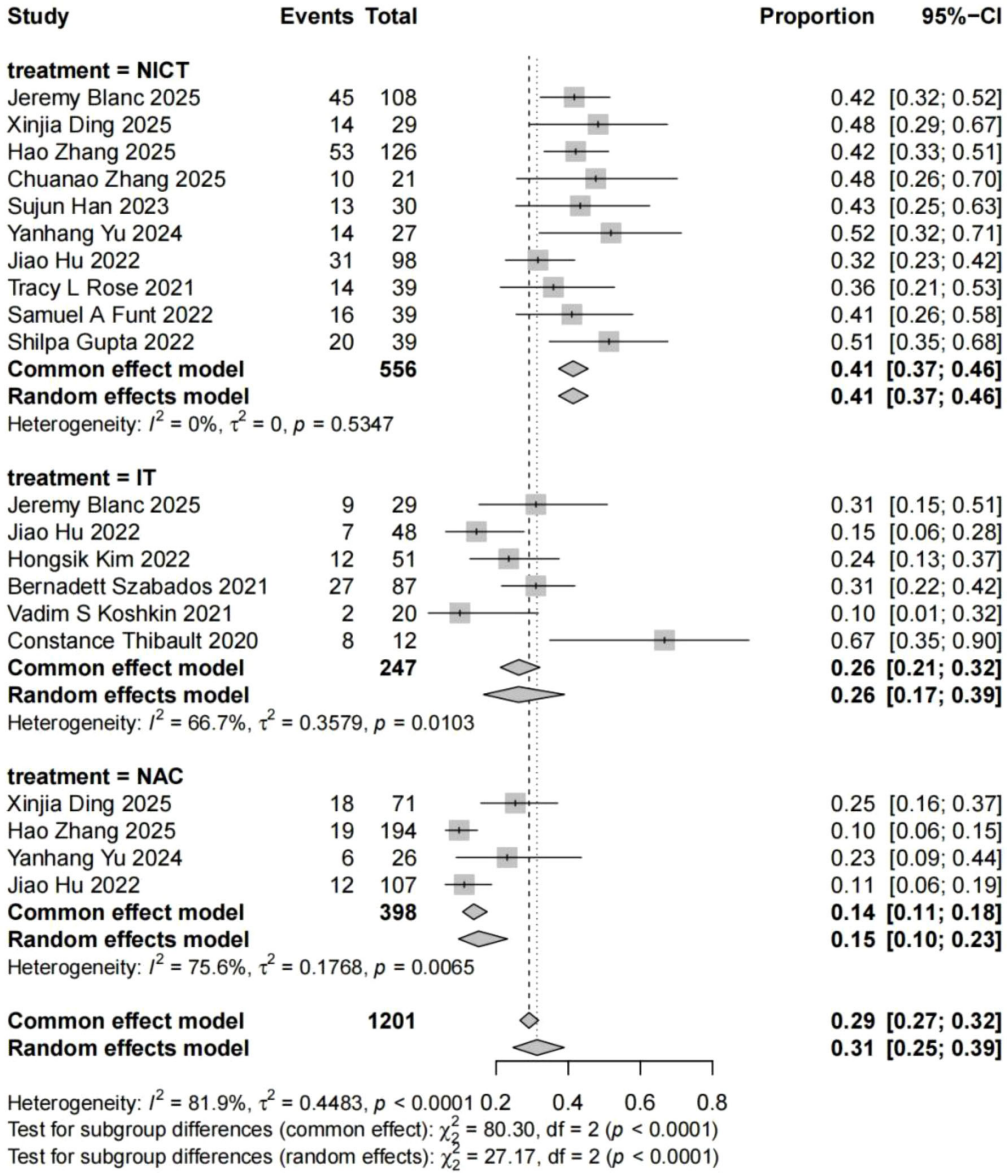
Forest plot of pCR rates.

**Figure 3** shows the forest plot for pCR. The overall pooled pCR rate for NICT was 0.41 (95% CI: 0.37-0.46), for IT was 0.26 (95% CI: 0.17-0.39), and for NAC was 0.15 (95% CI: 0.10-0.23).

**Figure 4:**
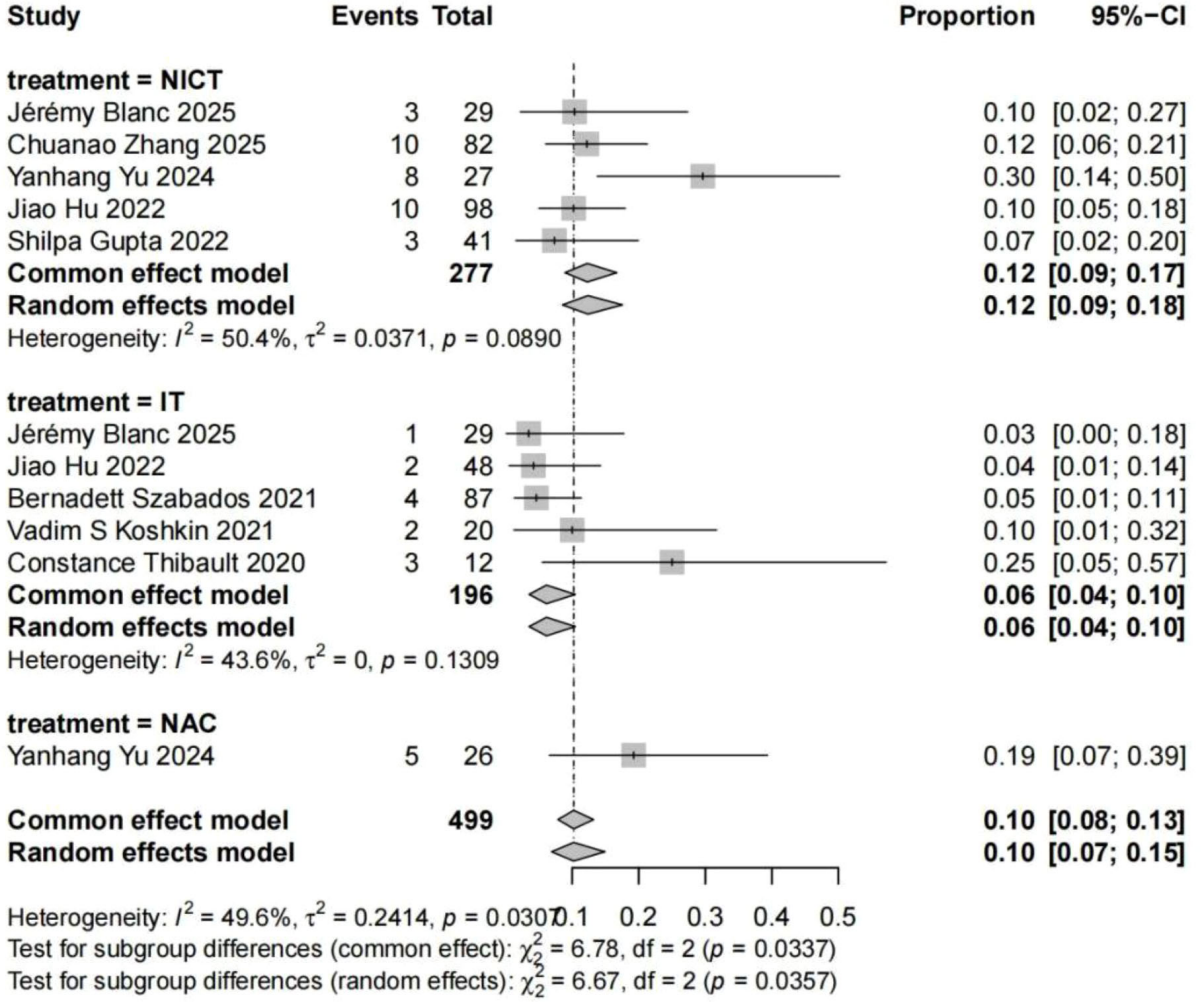
Forest plot of grade ≥3 AEs.

**Figure 4** shows the forest plot for grade ≥3 AEs. The highest pooled incidence was for NAC (0.19, 95% CI: 0.07-0.39), followed by NICT (0.12, 95% CI: 0.09-0.18), and IT (0.06, 95% CI: 0.04-0.10). Significant heterogeneity was observed in the NAC group.

### 3.4 Network graphs and consistency analysis

**Figure 5:**
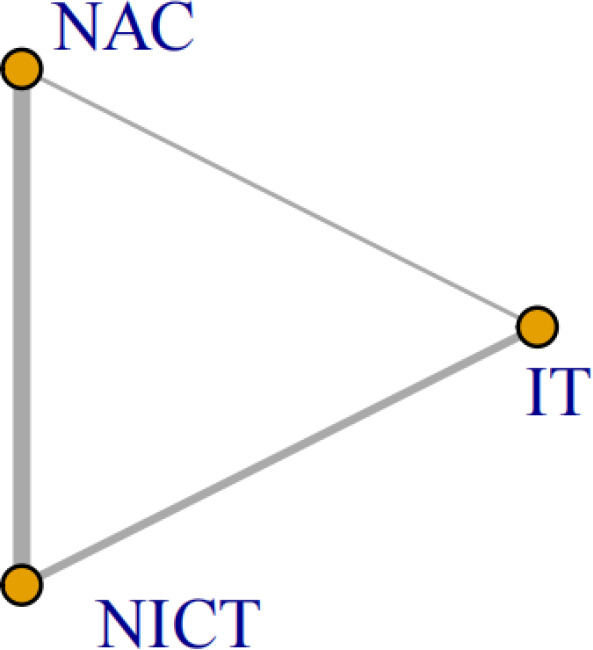
Network graph for efficacy.

**Figure 6:**
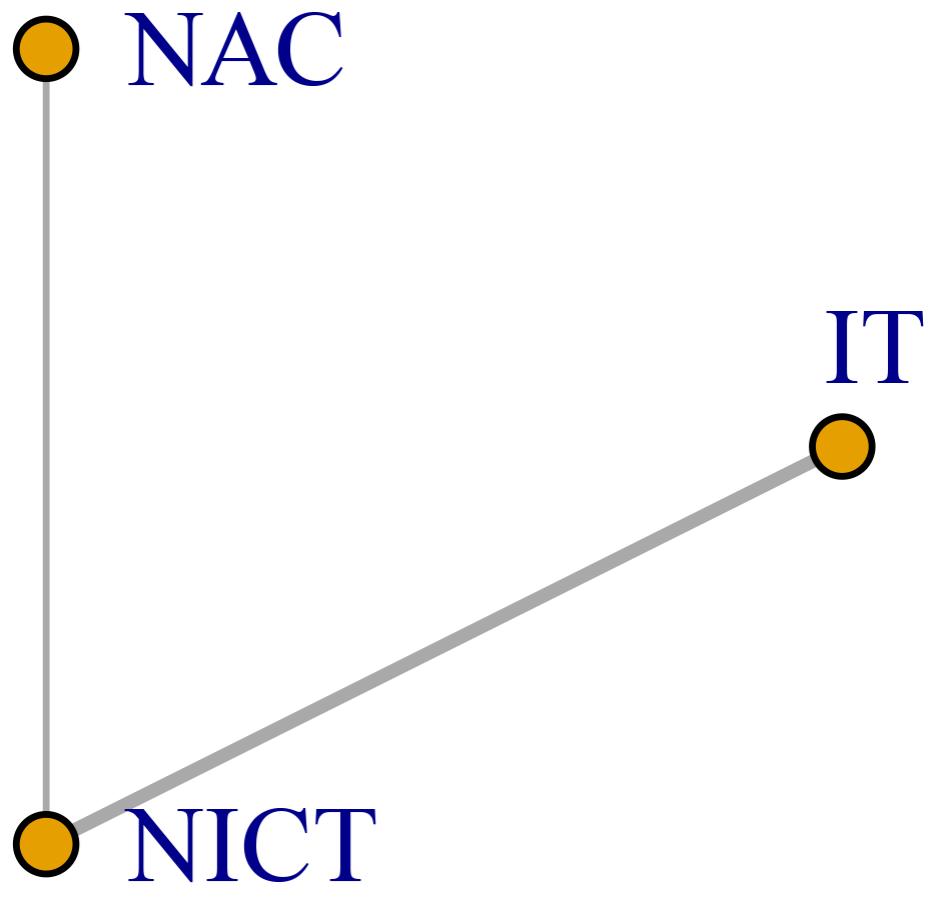
Network graph for safety.

Network graphs for the efficacy and safety comparisons are shown in **Figures 5**, **6**. For safety, no direct comparison between IT and NAC was available.

**Figure 7:**
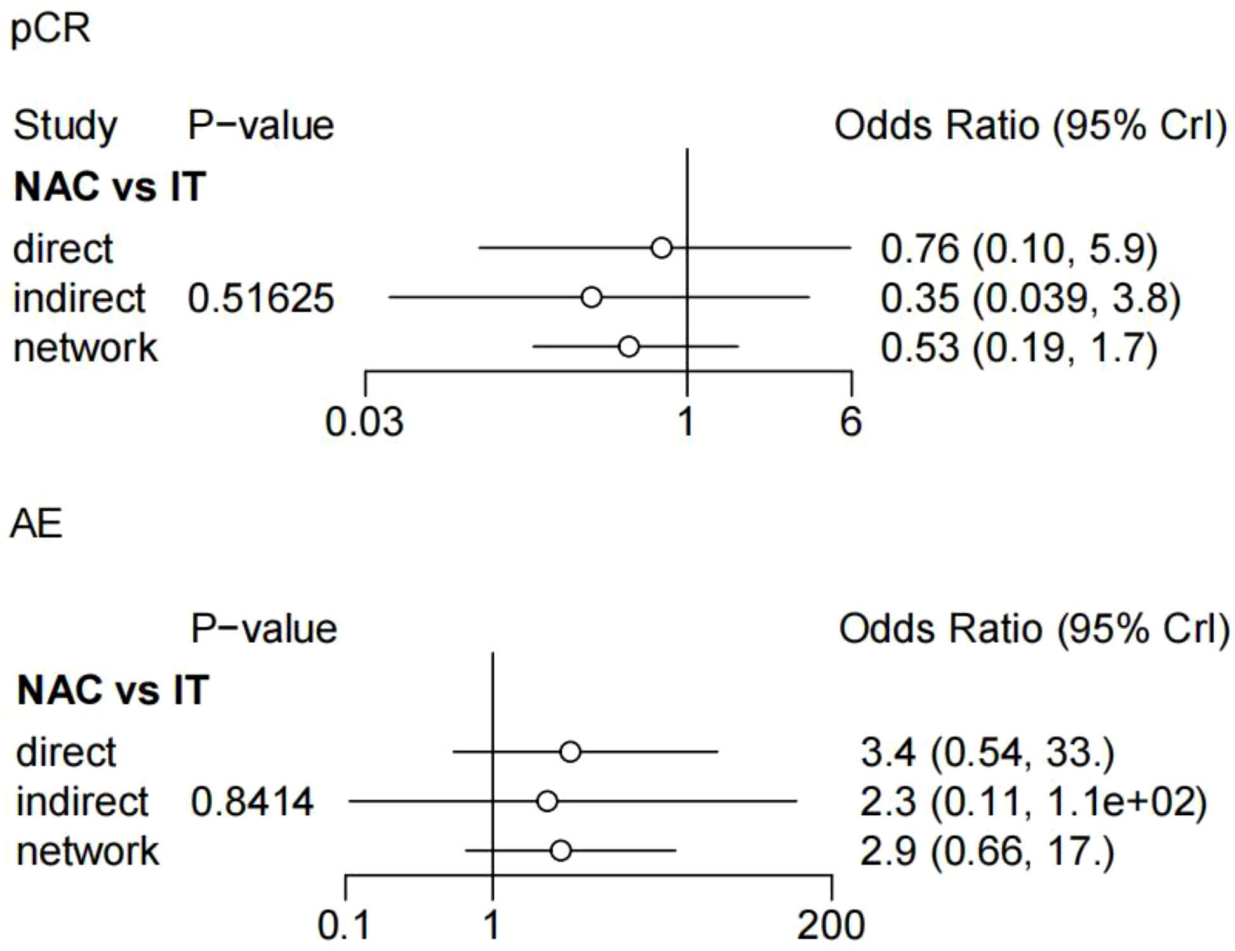
Consistency analysis plot.

Consistency analysis (**Figure 7**) showed no statistically significant difference between direct and indirect comparisons for NAC versus IT regarding pCR rate and AEs (P > 0.05), indicating good consistency and low heterogeneity.

### 3.5 Heterogeneity in network analysis

**Figure 8:**
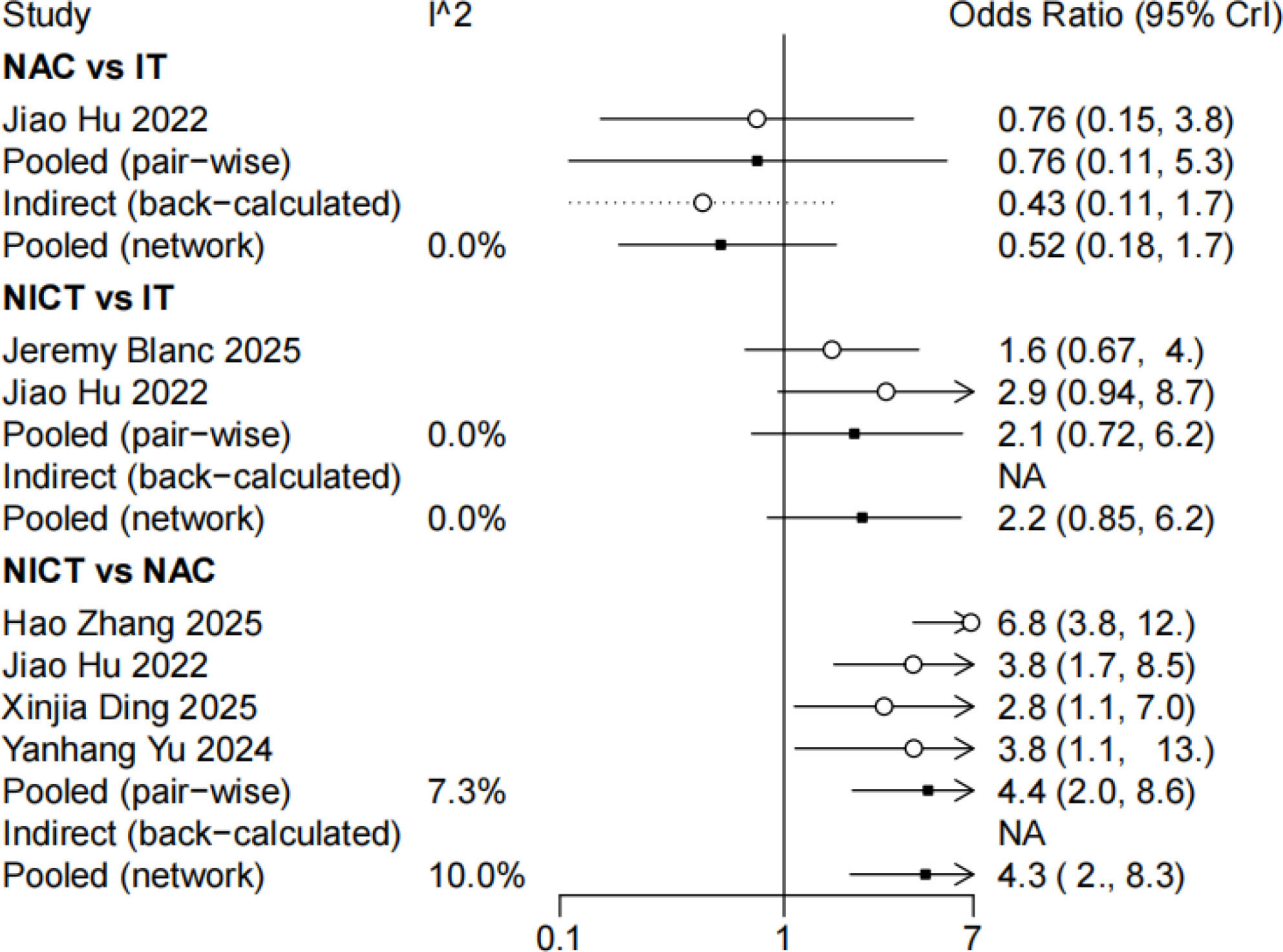
Heterogeneity assessment for the pCR endpoint.

**Figure 8** shows heterogeneity assessment for the pCR endpoint. I² was 0% for NAC vs. IT and NICT vs. IT, indicating no heterogeneity, while slight heterogeneity was present for NICT vs. NAC.

**Figure 9:**
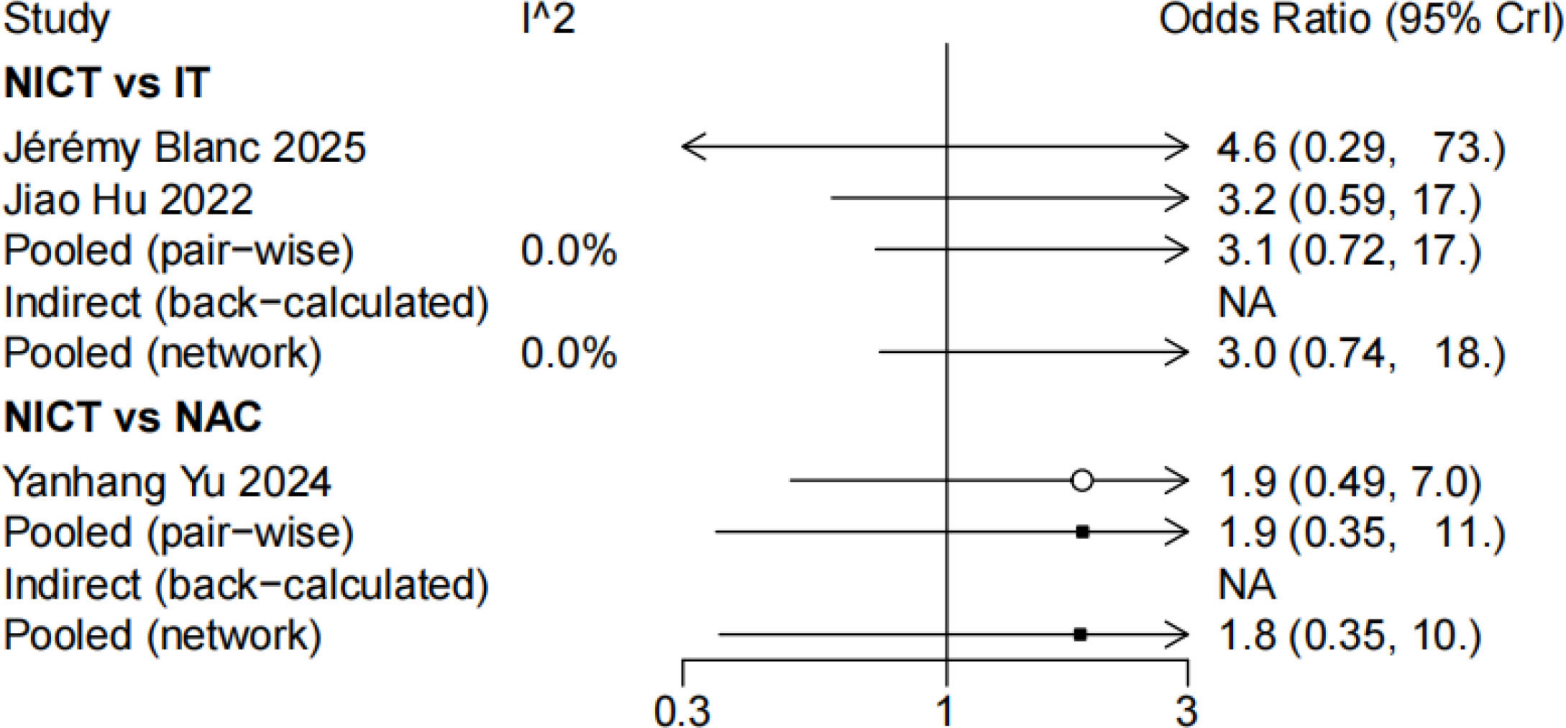
Heterogeneity assessment for the AE endpoint.

**Figure 9** shows heterogeneity assessment for the AE endpoint. No heterogeneity (I² = 0%) was found among the three groups.

### 3.6 Bayesian network meta-analysis results

**Figure 10:**
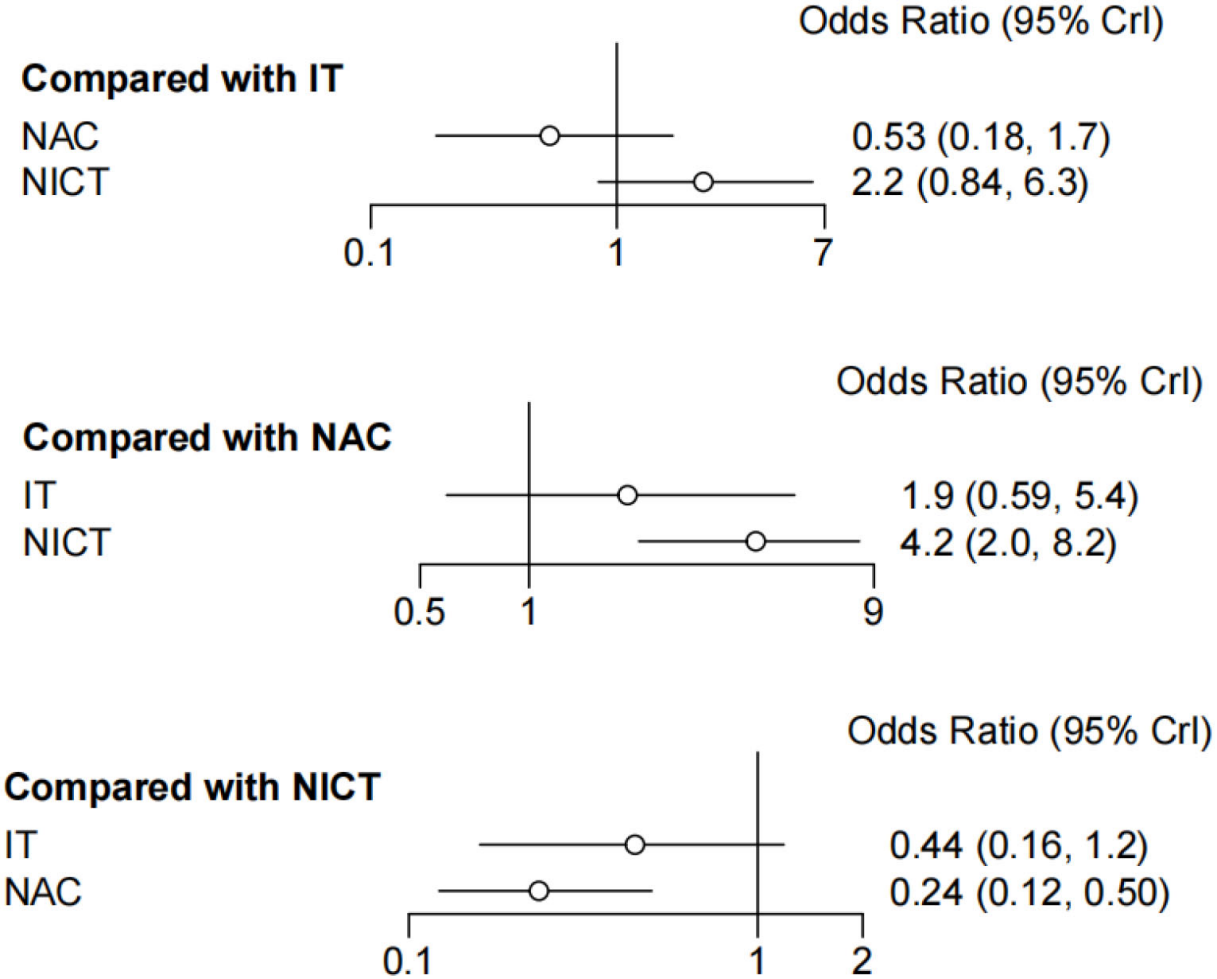
Forest plots from Bayesian network meta-analysis for efficacy.

**Figure 10** presents forest plots showing the efficacy comparisons using each treatment (IT, NAC, NICT) as the reference, expressed as OR and 95% CrI.

**Table 2** is the league table for efficacy comparison, showing pairwise ORs for pCR rates. NICT was significantly superior to NAC, while other comparisons showed no significant differences.

**Table 2:** League table for efficacy comparison.

Treatment	IT	NAC	NICT
IT	1.00	-0.63 (-1.69, 0.56)	0.81 (-0.17, 1.84)
NAC	0.63 ( -0.56, 1.69)	1.00	1.44 (0.68, 2.11)
NICT	-0.81 (-1.84, 0.17)	-1.44 (-2.11, -0.68)	1.00

**Table 3** is the league table for safety comparison, showing pairwise ORs for grade ≥3 AEs. No significant differences were observed between any treatment groups.

**Table 3:** League table for safety comparison.

Treatment	IT	NAC	NICT
IT	1.00	0.54 (-1.73, 2.91)	1.11 (-0.37, 2.80)
NAC	-0.54 (-2.91, 1.73)	1.00	0.59 (-1.07, 2.31)
NICT	-1.11 (-2.80, 0.37)	-0.59 (-2.31, 1.07)	1.00

**Figure 11:**
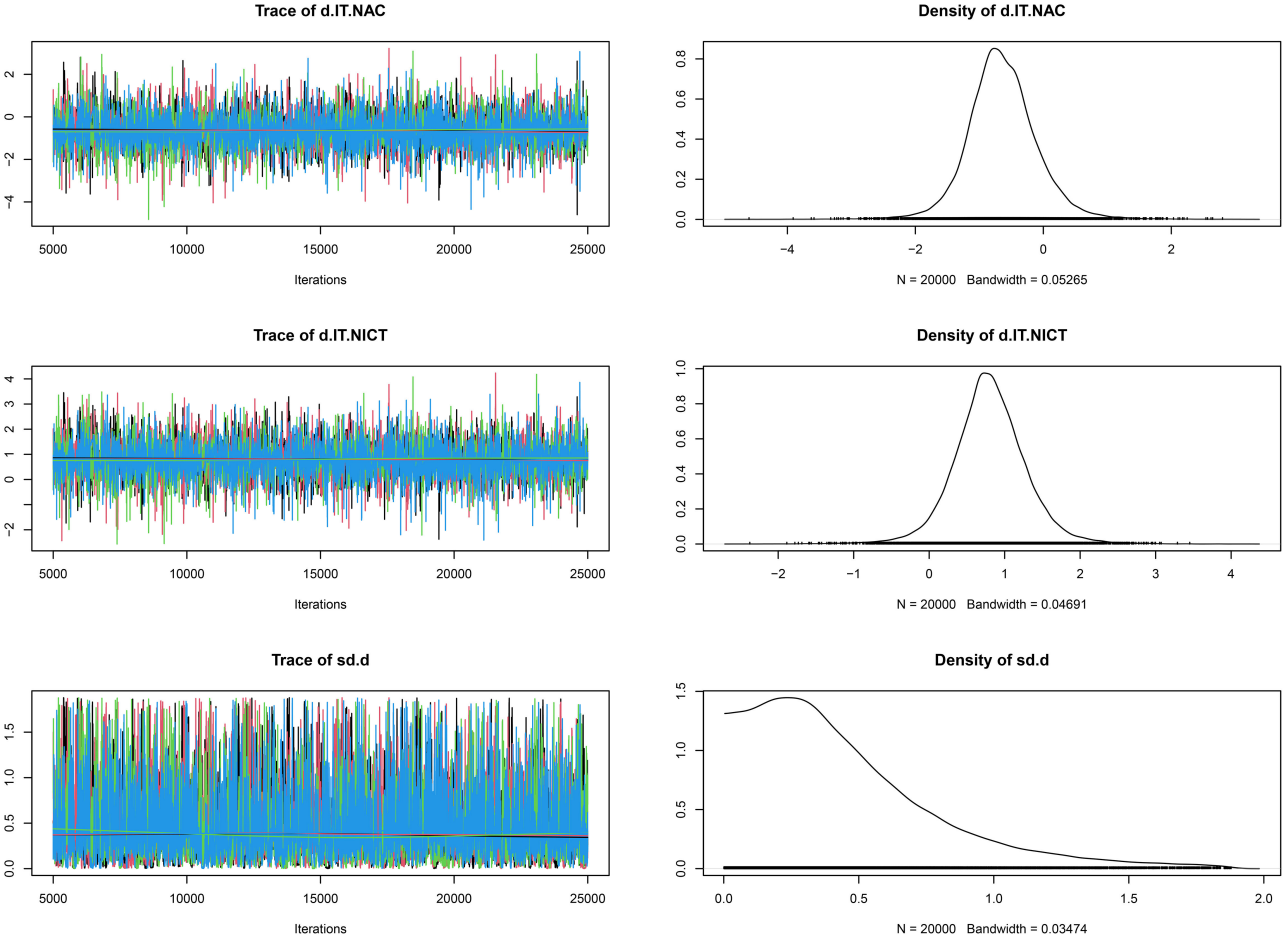
Trace plots for Bayesian network meta-analysis.

**Figure 12:**
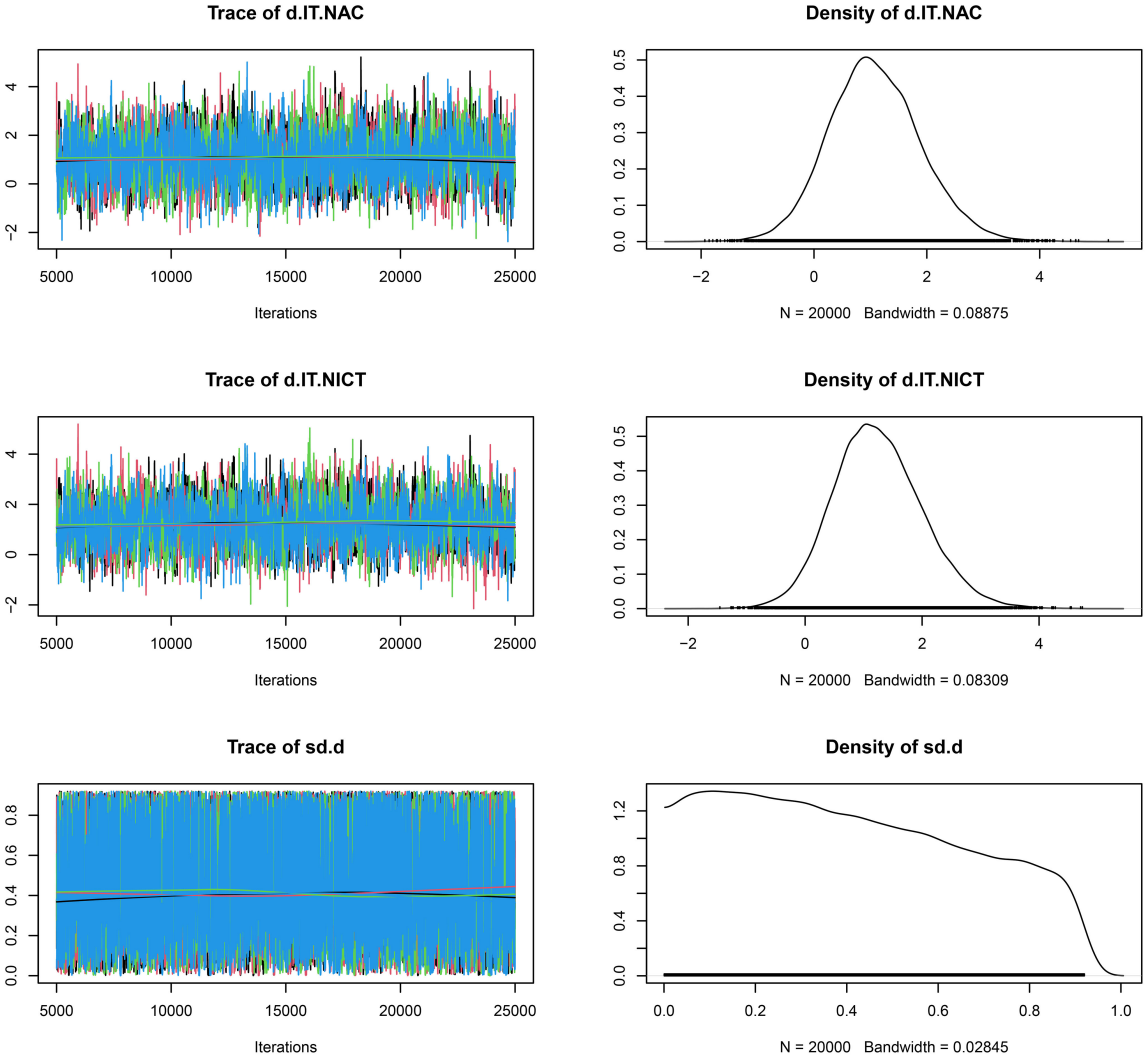
Density plots for Bayesian network meta-analysis.

**Figures 11**, **12** show the trace plots and density plots, respectively, from the Bayesian network meta-analysis for efficacy and safety. After 20,000 iterations, Markov Chain Monte Carlo trajectories stabilized with good overlap between chains, and kernel density bandwidths stabilized near zero. These results, along with non-significant node-splitting tests (all p > 0.05), support network consistency. Gelman-Rubin plots (**Figures 11**, **12**) showed excellent convergence (all Rê ≈1.0), indicating reliable parameter estimates.

**Figure 13:**
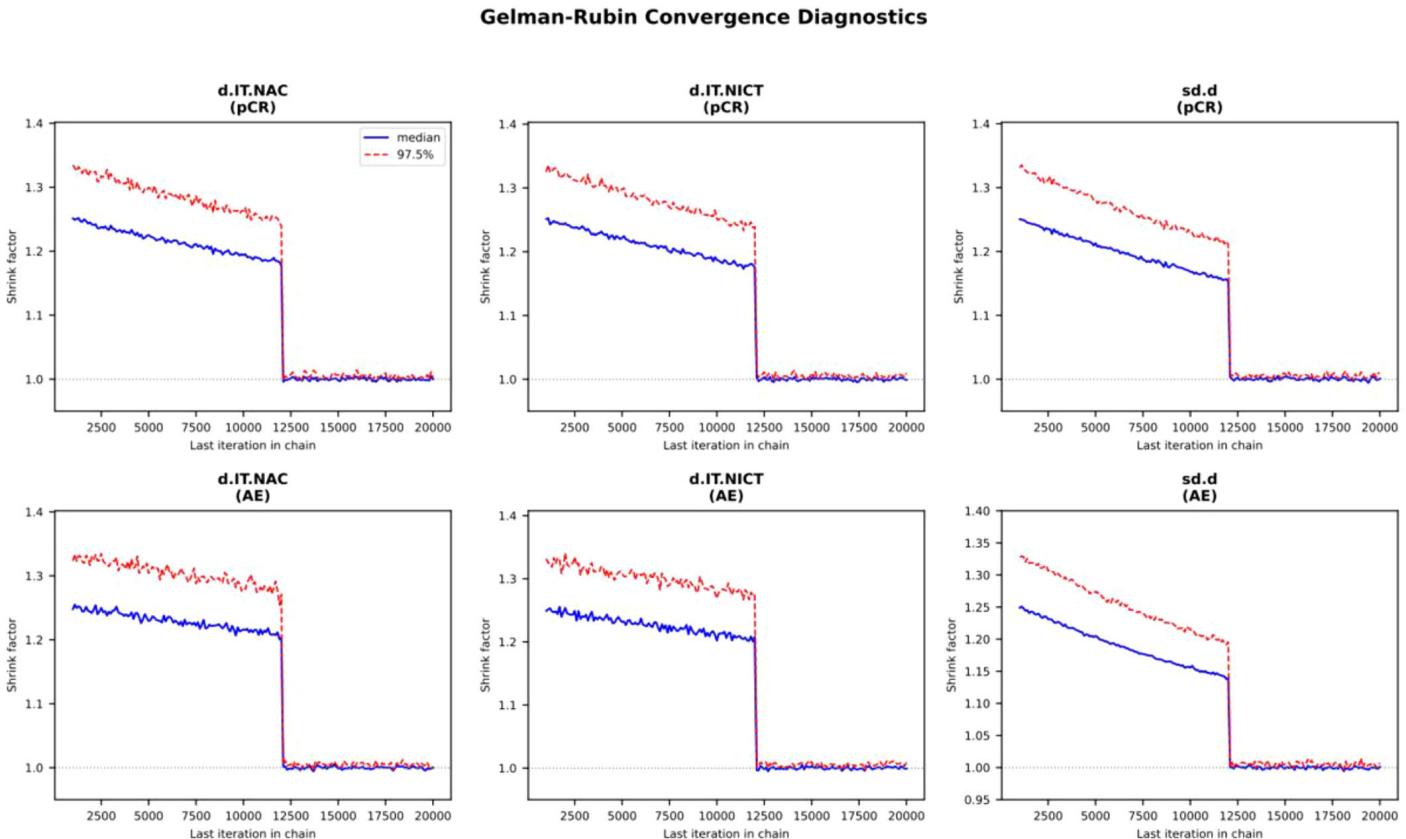
Gelman-Rubin convergence diagnostics for key model parameters.

Formal Gelman-Rubin convergence diagnostics for key model parameters are provided in **Figure 13**, confirming that all Rê values approached 1.0.

**Figure 14:**
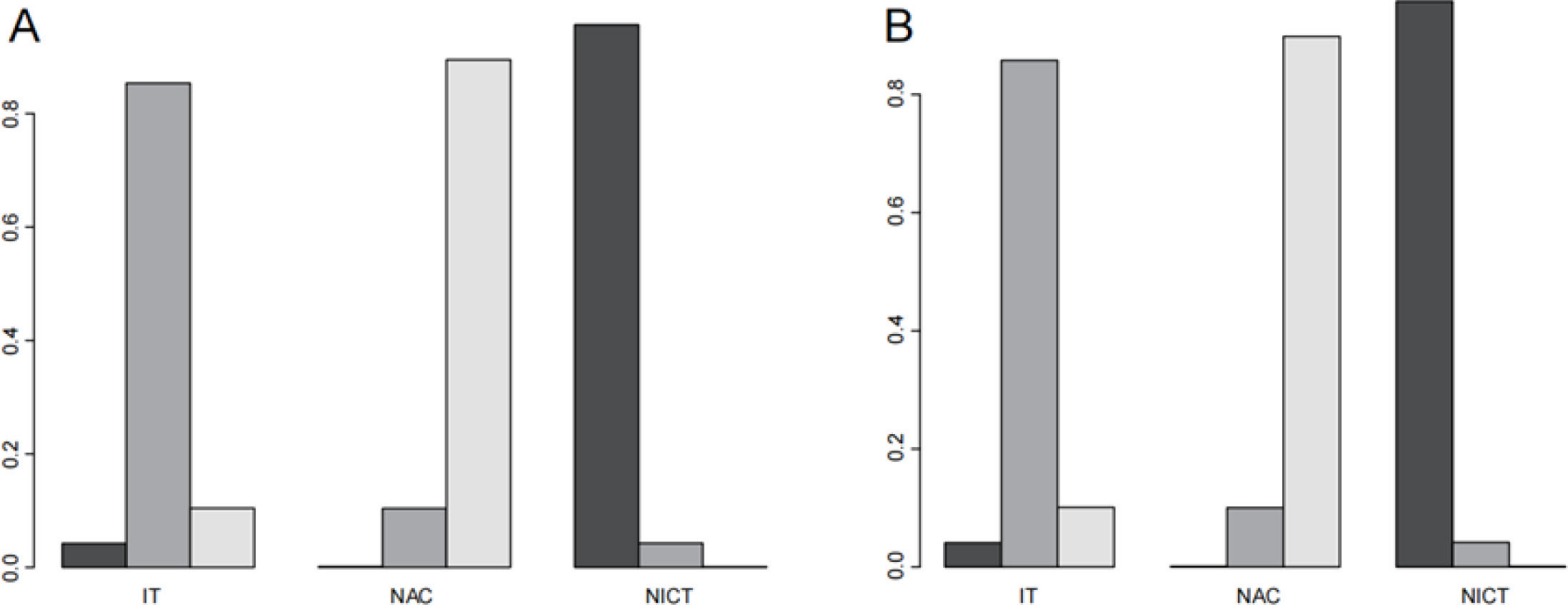
SUCRA ranking probabilities for efficacy **(A)** and safety **(B)**.

SUCRA rankings for the pCR endpoint were NICT (0.976) > NAC (0.519) > IT (0.471), suggesting NICT has the highest probability of being the most efficacious treatment (**Figure 14A**). For the AE endpoint, rankings were NICT (0.978) > IT (0.470) > NAC (0.051), suggesting NICT has the highest probability of adverse events (**Figure 14B**).

**Figure 15:**
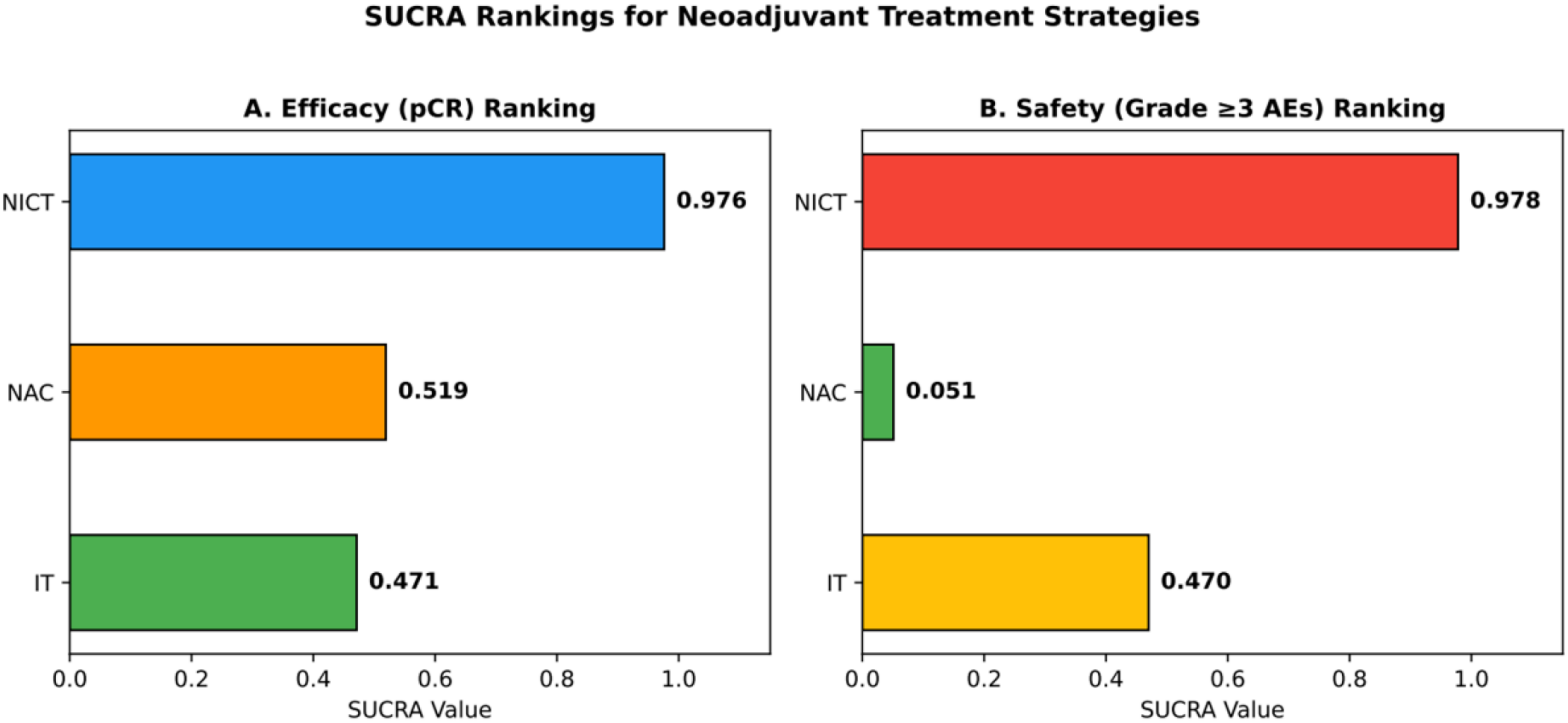
Detailed SUCRA ranking visualization for efficacy and safety. **(A)** SUCRA rankings for pathological complete response (pCR). **(B)** SUCRA rankings for grade ≥3 treatment-related adverse events (TRAEs).

A detailed visualization of the SUCRA rankings for both efficacy and safety is presented in **Figure 15**.

### 3.7 Sensitivity analysis

**Figure 16:**
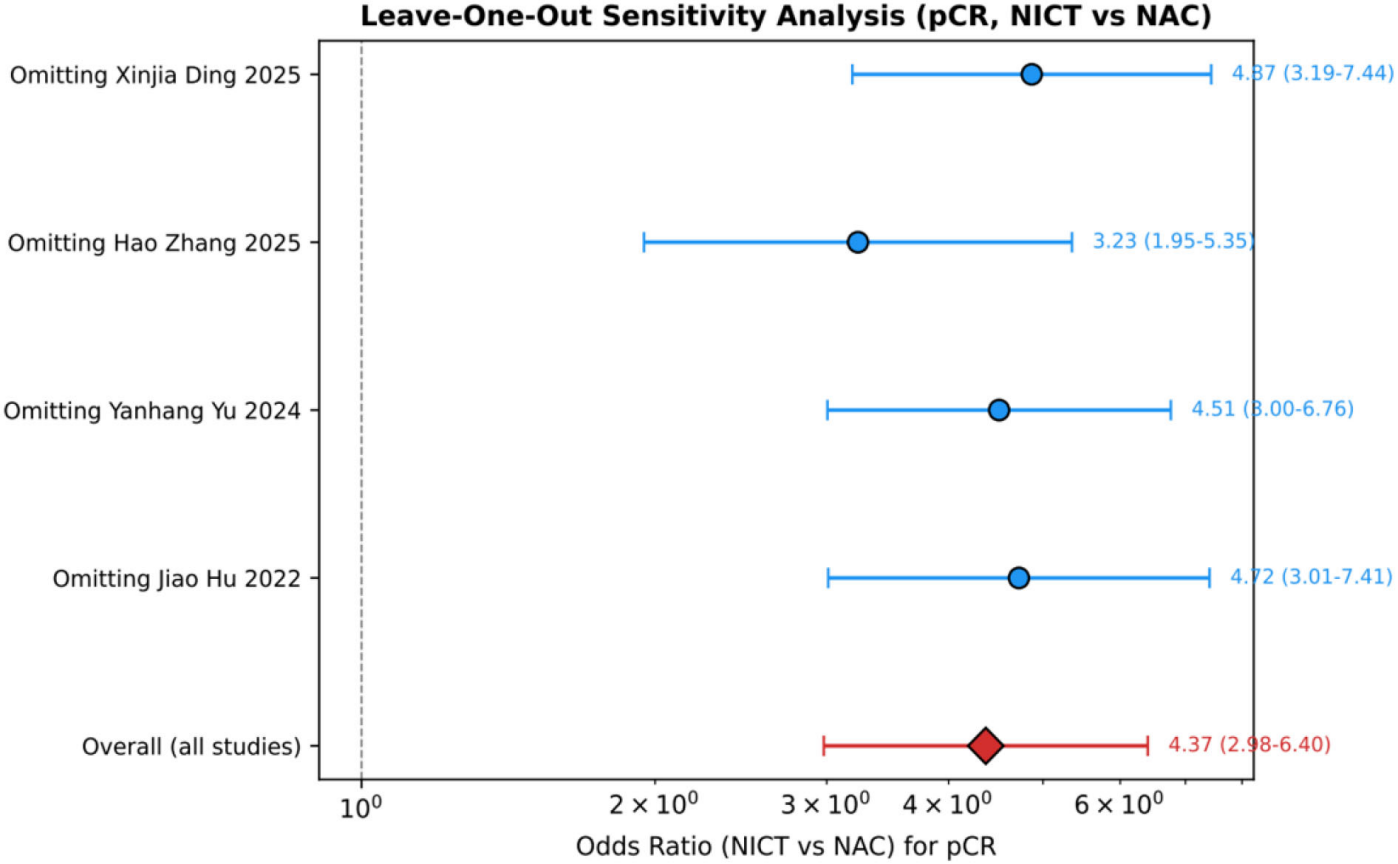
Leave-one-out sensitivity analysis for NICT versus NAC (pCR).

To assess the robustness of our findings, particularly regarding the inclusion of non-randomized studies, we performed a leave-one-out sensitivity analysis for the key comparison of NICT versus NAC. As shown in **Figure 16**, the pooled OR remained stable across all iterations, with no single study exerting disproportionate influence on the overall effect estimate.

### 3.8 Publication bias

**Figure 17:**
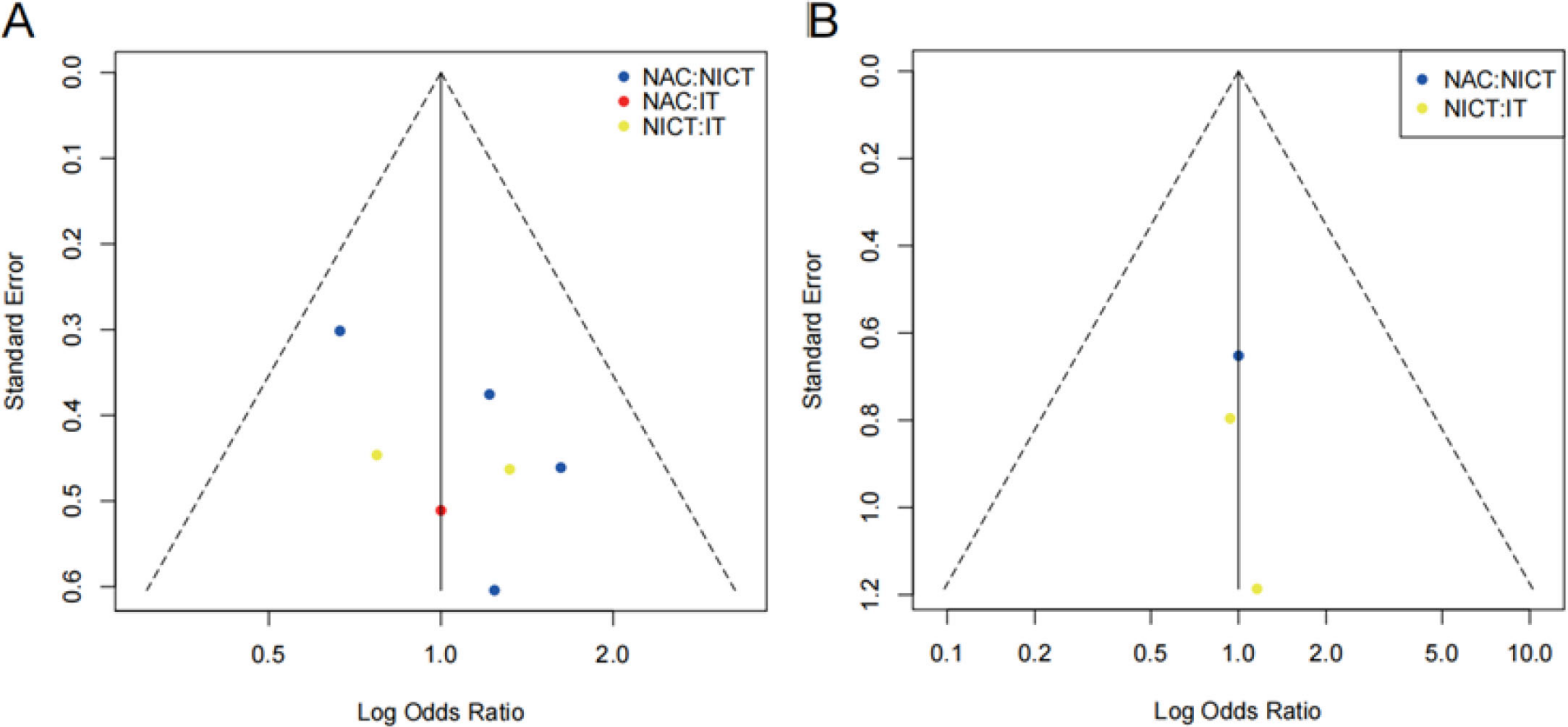
Funnel plots for publication bias assessment **(A)** pCR, **(B)** grade ≥3 AEs.

Funnel plots for the main outcomes showed some asymmetry (**Figure 17**), likely due to the limited number of studies and inherent heterogeneity rather than obvious publication bias, a conclusion supported by non-significant Egger’s tests (p = 0.12). Nevertheless, the asymmetry suggests that smaller studies may have reported more favorable outcomes for NICT, warranting cautious interpretation of its efficacy.

### 3.9 Efficacy-safety trade-off visualization

**Figure 18:**
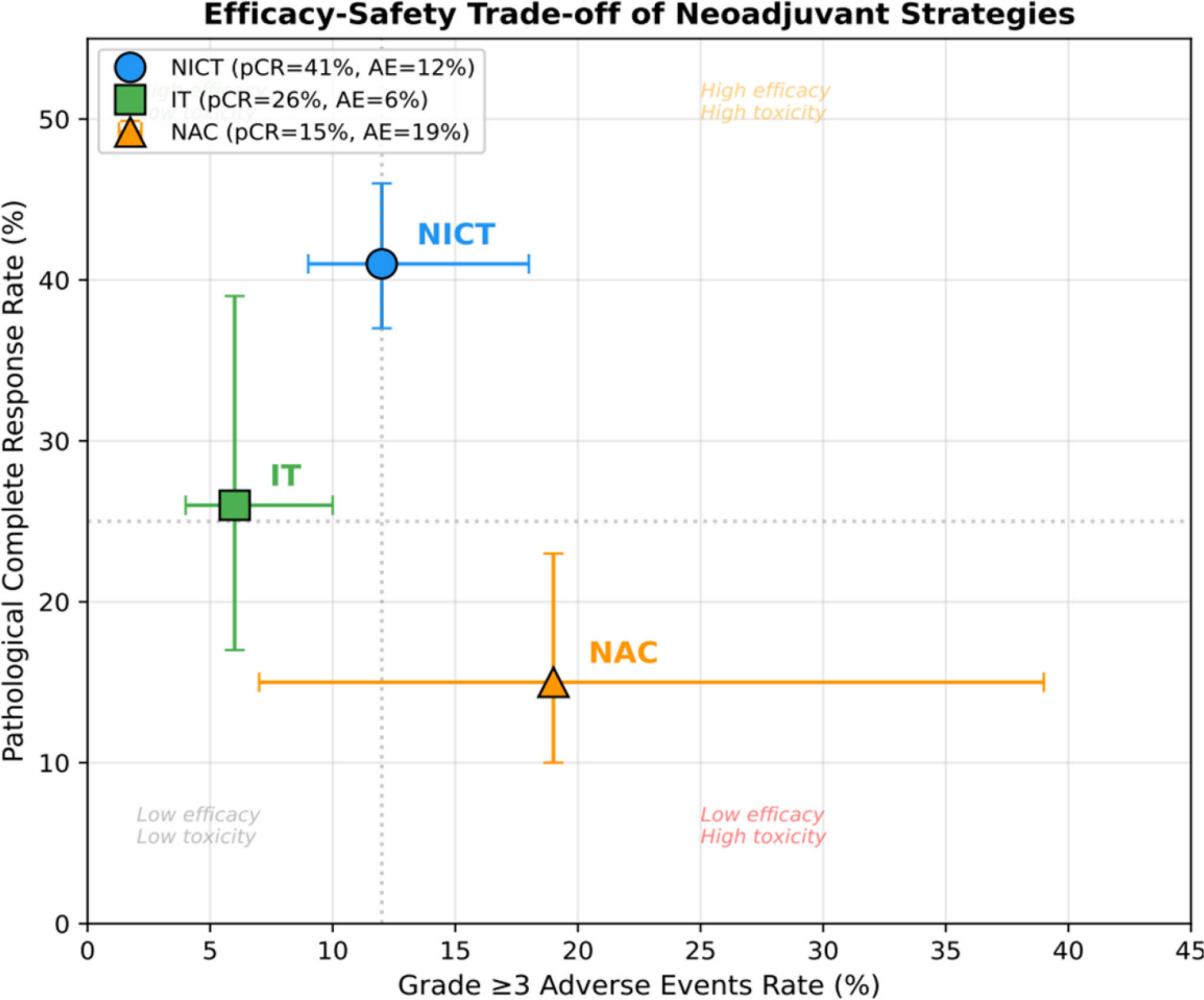
Efficacy-safety trade-off of neoadjuvant strategies. The x-axis represents the incidence of grade ≥3 TRAEs, and the y-axis represents the pCR rate. Error bars indicate 95% confidence intervals.

To facilitate clinical decision-making, we visualized the trade-off between efficacy (pCR rate) and safety(incidence of grade ≥3 TRAEs) for each treatment strategy. As shown in **Figure 18**, NICT offers the highest pCR rate but is associated with intermediate toxicity, whereas IT provides the safest profile with moderate efficacy, and NAC falls in between.

## 4 Discussion

By integrating data from the recently published phase III NIAGARA trial (19), our updated analysis provides the most current and comprehensive evidence in this field. Our findings corroborate and extend previous meta-analyses (16–18). While Matsukawa et al. (16) focused on immune checkpoint inhibitors in the neoadjuvant setting, and Hu et al. (18) examined PD-1/L1 inhibitors specifically, our study is the first to simultaneously rank NICT, NAC, and IT within a Bayesian framework, explicitly quantifying the efficacy-safety trade-off to guide individualized treatment selection. This Bayesian network meta-analysis is the first to systematically compare the efficacy and safety of NICT, NAC, and IT in MIBC. The key finding is that NICT leads to superior pCR rates compared to both NAC and IT. This aligns with results from trials like BLASST-1 (14), and the NIAGARA trial, which demonstrated a significant improvement in event-free survival for patients receiving perioperative durvalumab plus chemotherapy (19). suggesting NICT holds real promise in the neoadjuvant setting. For example, the phase II trial by Funt et al. reported a pCR rate of 41% for atezolizumab plus GC, compared to 27% for GC alone (12), suggesting a synergistic effect of the combination. That’s a clear “1 + 1>2” effect. The mechanism likely involves chemotherapy-induced tumor cell death releasing neoantigens, which in turn boosts T-cell infiltration and enhances the action of immune checkpoint inhibitors (36). Chemotherapy can also help by reducing immunosuppressive cells like Tregs and MDSCs, effectively ‘reprogramming’ the tumor microenvironment (37). Our use of SUCRA rankings within the Bayesian framework further solidifies this: NICT scored a high 0.976, clearly topping the list for efficacy. This points to NICT potentially reshaping the preoperative treatment landscape for MIBC and possibly improving long-term outcomes.

Interestingly, IT did not show a significant pCR advantage over NAC in this analysis (OR = 0.53). This might be down to the relatively small combined sample size for this comparison (n=275) or the variation in immunotherapy drugs and schedules across the included studies. For instance, the ABACUS trial using atezolizumab monotherapy reported a pCR of 31% (32), while PURE-01 using pembrolizumab monotherapy reported 37.5% (38), So, the specific drug matters, and this variability could dilute the apparent effect when studies are pooled.

However, this efficacy improvement must be considered in the context of patient-specific factors, such as PD-L1 expression levels, where patients with high expression may benefit more, underscoring the need for individualized patient selection (39). Our analysis found that NICT was associated with more grade ≥3 side effects compared to IT alone. While the direct pairwise comparisons in the league table didn’t reach statistical significance (possibly due to limited power), the SUCRA ranking clearly flags NICT as having the worst safety score. This makes biological sense – you’re combining the classic toxicities of chemotherapy (like bone marrow suppression and kidney issues) with the unique immune-related adverse events (irAEs) from checkpoint inhibitors (think colitis, hepatitis) (40). Nevertheless, the pooled incidence for NICT (12%) sits between that of NAC (19%) and IT (6%). This suggests that the combination does not simply multiply toxicity; careful management like dose adjustments (e.g., modified cisplatin dosing) and proactive AE management may mitigate this risk (35). The BLASST-1 trial, which used a split-dose cisplatin schedule with nivolumab plus GC, reported only 7% grade ≥3 TRAEs (14), illustrating this point.

Thus, a trade-off between efficacy and safety is evident. NICT achieved the highest SUCRA value for efficacy (0.978), whereas its AE incidence, though manageable, remains notable. Other studies have also found that the combination significantly boosts pCR without a dramatic spike in chemo-related toxicity, maintaining a safety profile that’s in the same ballpark as traditional regimens (41). The low overall heterogeneity (I²≈0%) in our analysis adds confidence to these pooled results. On balance, the combination seems to offer a meaningful efficacy advantage with a safety profile that, while requiring attention, isn’t prohibitive (28, 29). It underscores the importance of a multidisciplinary team to manage these patients effectively (42).

By using a network meta-analysis, we were able to make indirect comparisons between NICT and NAC/IT, filling a gap left by the lack of head-to-head randomized trials. Our finding of a 43% pCR for NICT, consistent with reports like Blanc et al. (26), reinforces that combination therapy is indeed more effective than immunotherapy alone (<15% in some studies). However, we should be mindful of potential sources of heterogeneity, like varying baseline PD-L1 expression across studies, which could influence effect estimates (16). A recent systematic review also confirmed NICT’s pCR advantage in real-world data but rightly highlighted that small sample sizes in many studies remain a limitation (17).

From a biological standpoint, the superior efficacy of NICT likely ties back to changes in the tumor microenvironment – chemotherapy-triggered cell death providing antigen, synergizing with PD-1/L1 blockade to ramp up CD8+ T cell activity (as explored by Ding et al., 2025). Notably, our pooled AE rate for NICT is lower than some real-world reports (e.g., Hu et al.’s multicenter data), suggesting potential selection bias in the literature – perhaps milder cases are more likely to be reported, potentially underestimating the true toxicity (31). Future research integrating deeper biomarkers, like tumor mutational burden (TMB) – which another meta-analysis linked to greater NICT benefit (18) – will be essential for identifying optimal candidates.

Our study has several strengths. First, this is the first NMA evaluating three neoadjuvant treatment strategies for MIBC, utilizing both direct and indirect evidence, providing a more comprehensive result. Second, considering the rapid evolution of oncology treatment paradigms, we included studies up to June 2025, ensuring the incorporation of the most recent trial data. Third, the entire analysis process strictly adhered to PRISMA-NMA guidelines, employing Bayesian models for robust inference and confirming convergence and consistency. Fourth, the inclusion of sensitivity analysis (leave-one-out) and convergence diagnostics (Gelman-Rubin) further strengthens the reliability of our findings.

Limitations should also be acknowledged. First, among the 14 studies, retrospective and single-arm designs were common, which can introduce selection bias. However, our leave-one-out sensitivity analysis (**Supplementary Figure 5**) suggested that no single study unduly influenced the pooled estimates, and the risk of bias assessment (**Supplementary Figure 3**) indicated moderate overall bias. Second, variations in the specific chemo/immuno agents, doses, and cycles across studies might affect the stability of our findings. For instance, most studies employed gemcitabine-based chemotherapy, while differences in cisplatin dosing schedules and the choice of PD-1/PD-L1 inhibitors could contribute to heterogeneity in both efficacy and safety outcomes. Third, we focused on short-term outcomes (pCR, TRAEs); data on long-term survival (OS, RFS) were lacking, which limits our ability to assess the durability of treatment benefits. Fourth, our analysis did not include emerging antibody-drug conjugate (ADC)-based regimens, such as EV + pembrolizumab. The phase III KEYNOTE-B15 trial has recently demonstrated that perioperative (neoadjuvant + adjuvant) EV + pembrolizumab significantly improves pCR (55.8% vs. 32.5%), event-free survival (HR 0.53), and overall survival (HR 0.65) compared with NAC in cisplatin-eligible patients with MIBC (4). This regimen is thus emerging as a new standard of care in the perioperative setting, although it has not yet been incorporated into major guidelines at the time of this analysis. Its exclusion from the present analysis represents a limitation, primarily because the primary results of KEYNOTE-B15 were presented after our literature search cutoff date (June 2025). For advanced or metastatic disease, the standard of care remains EV + pembrolizumab based on the KEYNOTE-302 trial (5). Future network meta-analyses should integrate this ADC-ICI combination to inform clinical decision-making accurately. The application of such combinations in the neoadjuvant setting is an active area of investigation, and their exclusion from the present analysis represents a limitation given the rapidly evolving treatment paradigm. Fifth, bladder-preserving approaches, including trimodal therapy (maximal transurethral resection followed by concurrent chemoradiotherapy), were not evaluated in this network meta-analysis, as all included studies required radical cystectomy as the definitive surgical treatment. Consequently, our findings are not generalizable to patients seeking bladder preservation. Finally, without patient-level biomarker data (e.g., PD-L1, TMB), we could not explore efficacy in specific subgroups. Future studies should integrate biomarker-based stratification to identify patients most likely to benefit from NICT or IT.

Looking ahead, we need larger, well-designed RCTs (like the ongoing phase III AURA trial) that include long-term survival endpoints. Combining this with deeper biologic profiling – think single-cell RNA sequencing – could help unravel why NICT works better for some and identify predictive biomarkers (TMB, immune cell density). Building predictive models and even exploring NICT combined with newer agents (like ADCs or CAR-T) are exciting future directions. The ultimate goal is to move away from a one-size-fits-all approach: identifying who truly needs the potency of NICT, who can do just as well with the safer IT option, and tailoring treatment precisely to the individual patient.

## 5 Conclusion

Our findings support a personalized approach to neoadjuvant therapy in MIBC. For patients with good performance status, cisplatin eligibility, and high tumor burden where maximal immediate response is desired, NICT appears to be the most effective option, albeit requiring careful toxicity management. For patients ineligible for cisplatin or with significant comorbidities limiting tolerance to combination therapy, IT offers a significantly safer alternative while still providing a meaningful pCR rate. NAC remains a standard for patients in an intermediate category, though our analysis suggests its efficacy is modest, especially in light of more potent combinations now available. Clinicians should be vigilant for hematologic toxicities with NAC and consider a switch to IT if significant AEs occur. In summary, NICT is most effective but carries higher risk; IT is safest with good efficacy; and NAC sits in the middle. The final choice should be guided by a careful assessment of each patient’s individual tolerance and risk profile.
