# Supplementary figures and images for: Choosing neoadjuvant therapy for muscle-invasive bladder cancer based on efficacy-safety trade-off: a network meta-analysis
Source: Front Oncol. 2026 Jun 12;16:1819312. doi: 10.3389/fonc.2026.1819312 (PMC13306761; doi:10.3389/fonc.2026.1819312)

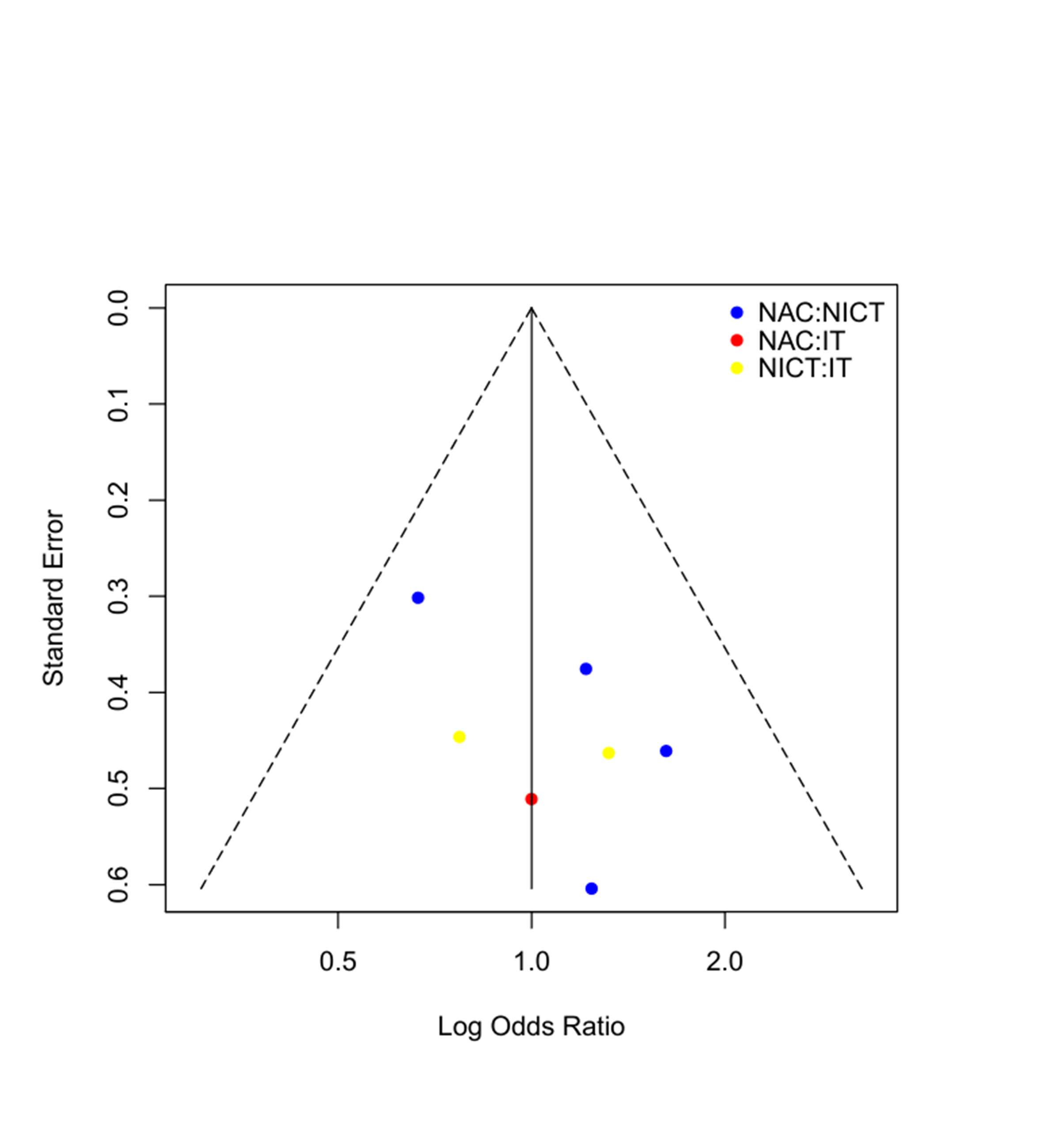

Supplement: Supplementary file 1 [file Image1.tif]

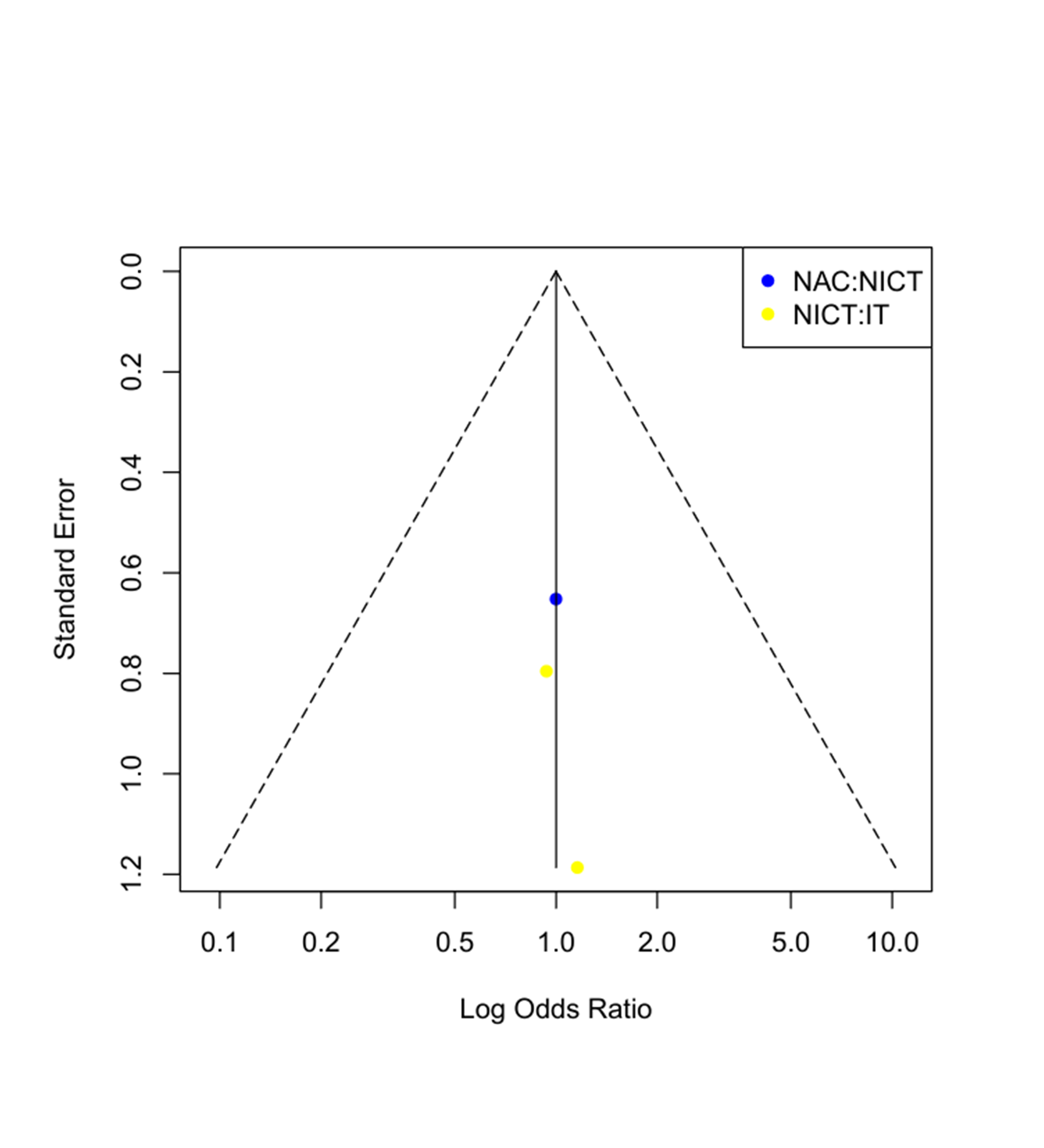

Supplement: Supplementary file 2 [file Image2.tif]
